# MicroRNA-214 protects against hypoxia/reoxygenation induced cell damage and myocardial ischemia/reperfusion injury via suppression of PTEN and Bim1 expression

**DOI:** 10.18632/oncotarget.13494

**Published:** 2016-11-22

**Authors:** Xiaohui Wang, Tuanzhu Ha, Yuanping Hu, Chen Lu, Li Liu, Xia Zhang, Race Kao, John Kalbfleisch, David Williams, Chuanfu Li

**Affiliations:** ^1^ Department of Surgery, James H. Quillen College of Medicine, East Tennessee State University, Johnson City, TN, USA; ^2^ Department of Geriatrics, First Affiliated Hospital with Nanjing Medical University, Nanjing, China; ^3^ Department of Biometry and Medical Computing, James H. Quillen College of Medicine, East Tennessee State University, Johnson City, TN, USA; ^4^ Center of Excellence for Inflammation, Infectious Disease and Immunity, James H. Quillen College of Medicine, East Tennessee State University, Johnson City, TN, USA

**Keywords:** microRNA-214, myocardial ischemia/reperfusion injury, myocardial apoptosis, PTEN, Bim1

## Abstract

**Background:**

Myocardial apoptosis plays an important role in myocardial ischemia/reperfusion (I/R) injury. Activation of PI3K/Akt signaling protects the myocardium from I/R injury. This study investigated the role of miR-214 in hypoxia/reoxygenation (H/R)-induced cell damage *in vitro* and myocardial I/R injury *in vivo*.

**Methods and Results:**

H9C2 cardiomyoblasts were transfected with lentivirus expressing miR-214 (LmiR-214) or lentivirus expressing scrambled miR-control (LmiR-control) respectively, to establish cell lines of LmiR-214 and LmiR-control. The cells were subjected to hypoxia for 4 h followed by reoxygenation for 24 h. Transfection of LmiR-214 suppresses PTEN expression, significantly increases the levels of Akt phosphorylation, markedly attenuates LDH release, and enhances the viability of the cells subjected to H/R. *In vivo* transfection of mouse hearts with LmiR-214 significantly attenuates I/R induced cardiac dysfunction and reduces I/R-induced myocardial infarct size. LmiR-214 transfection significantly attenuates I/R-induced myocardial apoptosis and caspase-3/7 and caspase-8 activity. Increased expression of miR-214 by transfection of LmiR-214 suppresses PTEN expression, increases the levels of phosphorylated Akt, represses Bim1 expression and induces Bad phosphorylation in the myocardium. In addition, *in vitro* data shows transfection of miR-214 mimics to H9C2 cells suppresses the expression and translocation of Bim1 from cytosol to mitochondria and induces Bad phosphorylation.

**Conclusions:**

Our *in vitro* and *in vivo* data suggests that miR-214 protects cells from H/R induced damage and attenuates I/R induced myocardial injury. The mechanisms involve activation of PI3K/Akt signaling by targeting PTEN expression, induction of Bad phosphorylation, and suppression of Bim1 expression, resulting in decreases in I/R-induced myocardial apoptosis.

## INTRODUCTION

MicroRNAs (miRs) are 21 to 23 nucleotide non-protein-coding RNA molecules, which have been identified as novel regulators of gene expression at the post-transcriptional level by binding to target messenger RNAs (mRNAs). Recently published data indicates that miRs, such as miR-1/106, miR-125b, miR-146a, miR-223, miR-21, miR-144/145, miR-320, miR-494, and miR-92a, are involved in ischemic heart disease [1–8]. miR-214 has been reported to protect cardiac myocytes from H_2_O_2_-induced injury [9]. Recently, Aurora *et al* [10] have shown that deficiency of miR-214 resulted in severe myocardial ischemia/reperfusion (I/R) injury and increased fibrosis progression as well as cardiac myocyte apoptosis. These authors demonstrated that miR-214 targets sodium-calcium exchanger-1, thus influencing cardiac myocyte calcium trafficking following myocardial I/R injury [10].

It is well known that myocardial apoptosis contributes to myocardial I/R injury [11]. Bad is a pro-apoptotic protein, a member of the Bcl-2 family and induces apoptosis by inhibiting the anti-apoptotic effects of Bcl-2 and Bcl-X, thereby allowing the pro-apoptotic proteins, Bak and Bax to aggregate and induce release of cytochrome c, followed by activation of caspase-mediated apoptotic signaling [12]. Phosphorylation of Bad by activated Akt prevents the interaction of Bad with Bcl-2 and Bcl-X [13]. Bcl-2 homology domain 3 (BH3)-only Pro-Protein Bim1 is an another pro-apoptotic protein and plays an important role in Bax/Bak mediated cytochrome c release and apoptosis [14]. Bim expression is regulated by activated FOXO3 (Forkhead box transcription factor, class O) which is controlled by activated phosphatidylinositol 3-kinase (PI3K)/Akt signaling [15]. In addition, activated Akt phosphorylates Bim1 at Ser87, resulting in blocking the pro-apoptotic effect of Bim1 [16].

Toll like receptor (TLR)-mediated innate immune and inflammatory responses have been demonstrated to play a critical role in myocardial ischemia/reperfusion (I/R) injury [17]. We have previously reported that administration of TLR ligands to mice induced protection against myocardial I/R injury [18–20]. The protective mechanisms involve the activation of PI3K/Akt signaling [18–21] which plays an important role in regulating cellular proliferation and survival [22, 23]. Phosphatase and tensin homolog (PTEN) is a tumor suppressor lipid protein phosphatase which negatively regulates PI3K/Akt signaling activation [24] by dephosphorylating PIP3 at its 3’ inositol position, resulting in decreased translocation of Akt to cellular membranes and subsequent down-regulation of PI3K/Akt activation [25]. Recent studies have shown PTEN plays a critical role in mitochondrial dependent apoptosis [26].

In the present study, we demonstrated that increased expression of miR-214 by transfection of the myocardium with lentivirus expressing miR-214 (LmiR-214) protects hearts from I/R injury. The mechanisms involve suppression of PTEN expression, leading to activation of PI3K/Akt signaling, induction of Bad phosphorylation, and targeting Bim1 expression, resulting in attenuation of I/R-induced myocardial apoptosis.

## RESULTS

### Increased miR-214 levels suppressed PTEN expression and increased Akt phosphorylation in H9C2 cardiomyoblasts

**Figure 1:**
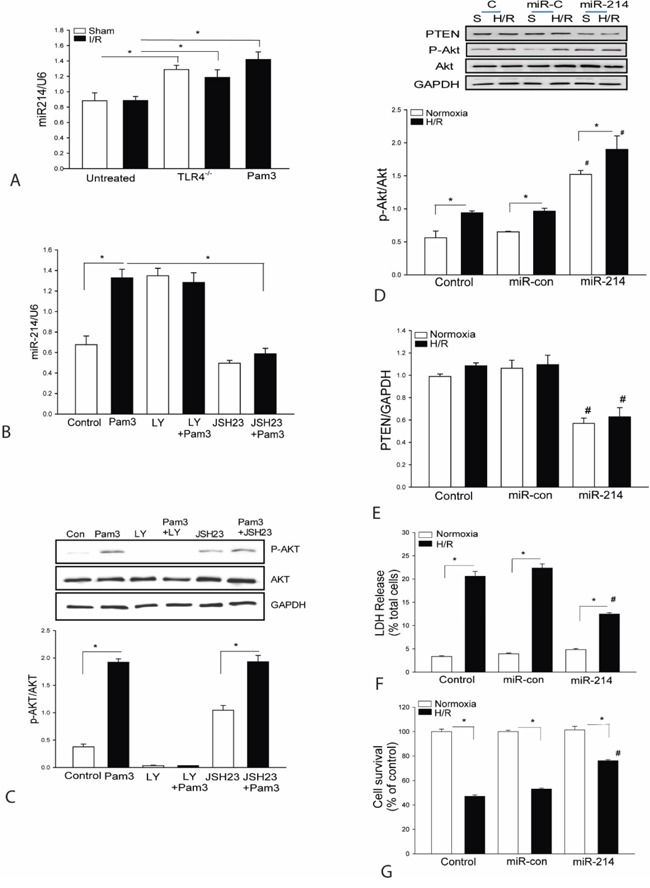
**A**. TLR4 deficiency or TLR2 ligand, Pam3CSK4 treatment increases the expression of miR-214 in the myocardium. TLR4 deficient (TLR4^-/-^) mice (n=3) or wild type (WT) mice were treated with and without Pam3CSK4 (50 μg/25 g body weight) and then subjected to myocardial ischemia (45 min) followed by reperfusion (4 h). Sham surgical operation served as sham control. Hearts were harvested and microRNAs were isolated for qPCR measurement of miR-214 (n=3-4/group). **B-C**. NF-κB activation is required for Pam3CSK4 induced miR-214 expression. Myoblast H9C2 cells were treated with PamsCSK4 in the presence of LY294002 or JSH23 for 24 hrs. The cells were harvested for qPCR measurement of miR-214 expression (B) and for Western blot analysis of Akt phosphorylation (C). There were 3 replicates in each group. * p<0.05 compared with indicated groups. **D-G**. H9C2 cells were transfected with lentivirus expressing miR-214 (LmiR-214) or lentivirus expressing miR-control (LmiR-control) respectively. The cells were subjected to hypoxia (4 h) followed by reoxygenation (24 h). The levels of Akt phosphorylation (D) and PTEN expression (E) were assessed by Western blot (n=3). LDH activity (F) in the supernatants was measured by a commercially available kit and cell viability (G) was measured by MTT assay (n=3-5). *p<0.05 compared with indicated groups. # p<0.05 compared with respective control.

We have previously reported that either TLR4 deficiency or TLR2 modulation by Pam3CSK4 significantly attenuates-I/R induced myocardial injury via activating PI3K/Akt dependent mechanism [20]. Interestingly, we observed that the levels of myocardial miR-214 were markedly greater in either TLR4 deficient mice or Pam3CSK4 treated mice compared with untreated group (Figure 1A). Our previous studies have shown that TLR2 modulation can significantly attenuate I/R induced myocardium injury by activating PI3K/Akt signaling. PTEN, a negative regulator of PI3K/Akt signaling is a potential target of miR-214. To investigate the underlying mechanisms by which TLR2 modulation regulates the miR-214 expression, H9C2 cardiomyoblasts were treated with TLR2 specific ligand Pam3CSK4. As shown in Figure 1B, the levels of miR-214 in Pam3CSK4 treated cells are significantly increased. The levels of phosphorylated Akt are also significantly increased following Pam3CSK4 treatment (Figure 1C) which is consistent with our previous studies. However, PI3K inhibition with LY294002 (LY) significantly prevented Pam3CSK4 induced Akt phosphorylation but did not alter Pam3CSK4 induced increases in miR-214 expression. To determine whether NF-κB signaling involves Pam3CSK4 induced miR-214 expression, we treated cells with NF-κB specific inhibitor, JSH-23 and observed that JSH-23 treatment significantly prevented Pam3CSK4 induced increases in the expression of miR-214 (Figure 1B). Collectively, these data suggest the TLR2 ligand induced increases in the expression of miR-214 are mediated through NF-κB activation pathway.

To examine whether increased expression of miR-214 will activate PI3K/Akt signaling, we transfected H9C2 cells with lentivirus expressing miR-214 (LmiR-214) or LmiR-control respectively, before the cells were subjected to hypoxia (4 h) followed by reoxygenation (H/R). Figures 1D and 1E show that LmiR-214 transfection markedly increases the levels of Akt phosphorylation and suppresses PTEN expression in the presence or absence of H/R. The data suggests that miR-214 targets PTEN expression, resulting in activation of PI3K/Akt signaling.

### Increased expression of miR-214 attenuates H/R induced cell injury and increases survival in H9C2 cardiomyoblasts

Activation of PI3K/Akt signaling plays an important role in protection against H/R-induced cell injury [27, 28]. We examined whether transfection of LmiR-214 will protect the H9C2 cells from H/R-induced injury. Figure 1F shows that H/R significantly increased LDH activity by 5.6-fold compared with the control cells (normoxia). In contrast, transfection of cells with LmiR-214 markedly attenuates H/R-induced LDH activity by 54%, when compared with untransfected H/R group. H/R also significantly decreased cell viability by 47% compared with normoxia group (Figure 1G). However, the viability in LmiR-214 transfected cells that were subjected to H/R was significantly greater by 75% compared with H/R group. Transfection of LmiR-control did not affect H/R-induced cell injury and death in H9C2 cells.

### LmiR-214 transfection attenuates cardiac dysfunction and reduces infarct size after myocardial I/R

**Figure 2:**
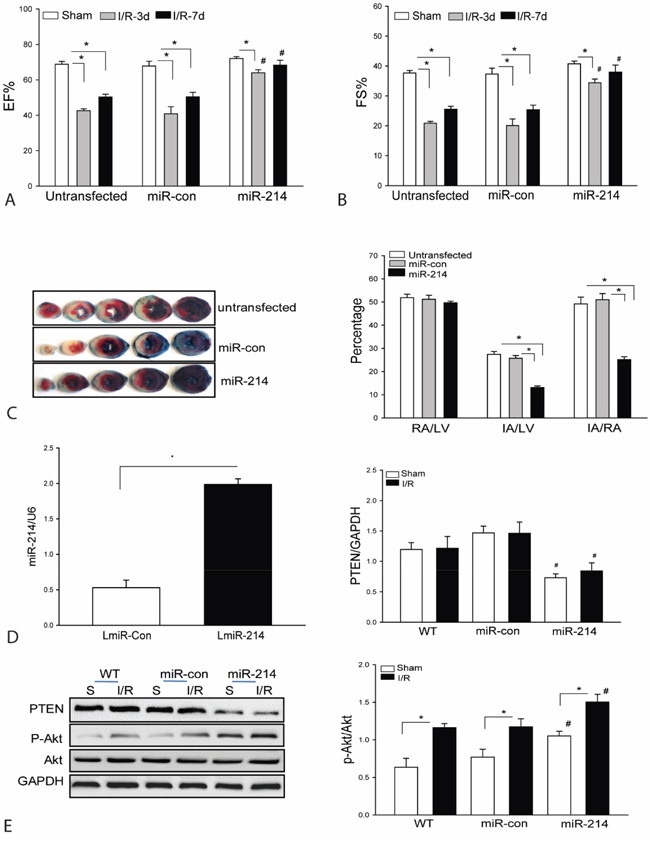
Transfection of lentivirus expressing miR-214 into the myocardium improves cardiac function and decreases infarct size following myocardial I/R injury Mouse hearts were transfected with LmiR-214 through the right common carotid artery (n=8/group). Seven days after transfection, hearts were subjected to ischemia (45 min) followed by reperfusion for up to 7 days. **A** and **B**. Cardiac function was measured by echocardiography 3 and 7 days after myocardial I/R. **C**. Hearts were harvested 24 h after reperfusion for TTC staining infarct size. **D**. The level of miR-214 was increased following LmiR-214 transfection. **E**. LmiR-214 transfection suppresses PTEN expression and increases Akt phosphorylation levels. n=6/group. * P<0.05 compared with indicated group. # p<0.05 compared with respective control.

Our *in vitro* data shows that increased expression of miR-214 markedly activates PI3K/Akt signaling and protects H9C2 cells from H/R-induced injury. We evaluated whether *in vivo* transfection of LmiR-214 will protect the heart from myocardial I/R injury. Mouse hearts were transfected with LmiR-214 through the right carotid artery [1, 2]. LmiR-control served as vector control. Figure 2D shows that seven days after transfection, the levels of miR-214 in the myocardium were significantly increased by 3.7-fold compared with control. We also examined whether increased expression of miR-214 will protect against myocardial I/R-induced injury. The hearts were subjected to ischemia (45 min) followed by reperfusion up to 7 days. As shown in Figures 2A and 2B, I/R significantly decreased cardiac function. The values for ejection fraction (EF%) and fractional shortening (FS%) were markedly reduced by 38.1% and 44.5% on day 3 and by 24.6% and 32.1% on day 7 respectively, after myocardial I/R injury compared with sham control. In contrast, LmiR-214 transfection attenuated I/R-induced cardiac dysfunction. The values for EF% and FS% in LmiR-214 transfected hearts were significantly greater than in the untransfected I/R group. Figure 2C shows that transfection of LmiR-214 into the hearts markedly reduced infarct size by 52.1% compared with the untransfected I/R group. Transfection of LmiR-control into the myocardium did not alter I/R-induced decreases in the values of EF% and FS% and myocardial infarct size.

### LmiR-214 transfection suppresses PTEN expression and increases Akt phosphorylation in the myocardium

PTEN is a negative regulator of PI3K/Akt signaling [29, 30]. We examined the effect of LmiR-214 transfection on PTEN expression and Akt phosphorylation in the myocardium following I/R. Figure 2E shows that I/R did not affect PTEN expression but enhanced the levels of Akt phosphorylation compared with sham control. However, LmiR-214 transfection significantly suppresses the expression of PTEN and further increases Akt phosphorylation levels in both sham and I/R groups when compared with untransfected respective controls. In the LmiR-214 transfected group, the levels of phosphorylated Akt were increased by 34.1% and PTEN decreased by 42.4%, when compared with untransfected I/R hearts. Transfection of LmiR-control did not alter the levels of Akt phosphorylation and PTEN expression in the myocardium of both sham and I/R groups.

### Increased expression of miR-214 attenuates I/R induced myocardial apoptosis

**Figure 3:**
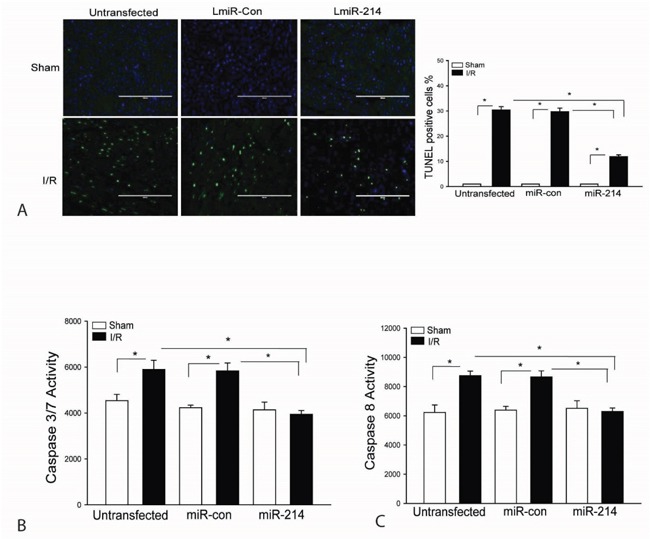
Increased expression of miR-214 attenuates I/R-induced myocardial apoptosis LmiR-214 or LmiR-control was transfected into the myocardium of mice via the right common carotid artery 7 days before the hearts were subjected to ischemia (45 min) followed by reperfusion (24 h). Hearts were harvested and sectioned for TUNEL assay of myocardial apoptosis **A**. Caspase-3/7 **B**. and Caspase-8 **C**. activities were measured by ELISA kits. N=4-6, *p<0.05 compared with indicated group.

It is well known that myocardial apoptosis contributes to cardiac dysfunction after myocardial I/R injury [31]. We examined the effect of LmiR-214 transfection on myocardial apoptosis following myocardial I/R injury. Figure 3A shows that I/R increased Tunnel positive myocardial apoptotic cells by 29.8% compared with sham control. In contrast, transfection of LmiR-214 into the myocardium significantly decreases I/R-induced myocardial apoptosis by 60.9%, when compared with the untransfected I/R group. Activation of caspase-3/7 and caspase-8 have been considered as specific markers for apoptosis [32, 33]. Figures 3B and 3C show that I/R-induced an increase in capase-7 by 30% and caspase-8 by 40.4%, when compared with sham control. However, increased expression of miR-214 prevents I/R-induced myocardial caspase-3/7 and caspase-8 activities (Figures 3B and 3C). Transfection of LmiR-control did not alter I/R-induced myocardial apoptosis.

### LmiR-214 transfection increases Bad phosphorylation and suppresses the expression of Bim1 in the myocardium

**Figure 4:**
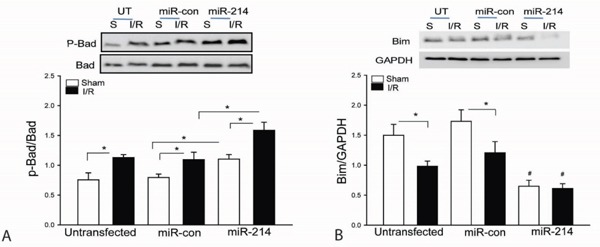
Increased expression of miR-214 suppresses Bim expression and increases Bad phosphorylation in the myocardium following myocardial I/R LmiR-214 or LmiR-control was transfected into the myocardium of mice via the right common carotid artery 7 days before the hearts were subjected to ischemia (45 min) followed by reperfusion (24 h). The hearts were harvested for isolation of cellular proteins. The levels of phosphorylated Bad **A**. and Bim1 **B**. were examined by Western blot. N=4-6, *p<0.05 compared with indicated group.

Bad is a pro-apoptotic protein which interacts with Bcl2, resulting in blocking Bcl2 anti-apoptotic function [34]. When Bad is phosphorylated, Bcl2 will release from Bad/Bcl2 complex and functions in an anti-apoptotic role [13, 34, 35]. Figure 4A shows that transfection of LmiR-214 markedly increases the levels of phosphorylated Bad in both sham and I/R groups compared with untransfected respective controls. Bim1 is a mitochondrial pro-apoptotic factor which will translocate from the cytosol to mitochondria, resulting in cardiomyocyte apoptosis during I/R [14, 36]. As shown in Figure 4B, I/R decreased the cytosolic levels of Bim1 compared with sham control. However, transfection of LmiR-214 further decreases the cytosolic levels of Bim1 in both sham and I/R groups, indicating increased expression of miR-214 may suppress the expression and translocation of Bim1 from cytosol to mitochondria. Transfection of LmiR-control did not alter I/R induced changes of Bad phosphorylation and Bim1 expression.

### LmiR-214 suppresses the translocation of Bim1 from cytosol to mitochondria in cardiomyoblasts following H/R

**Figure 5:**
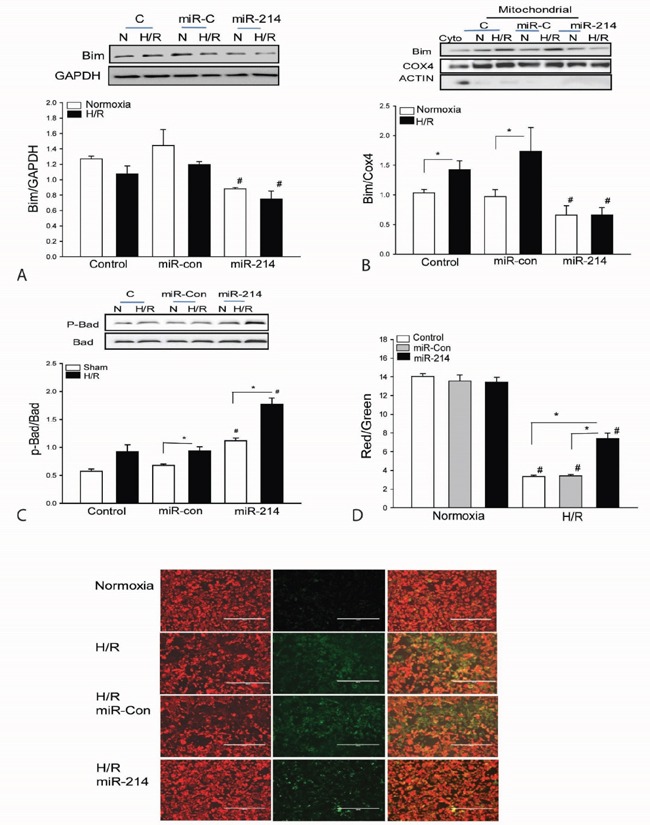
Increased expression of miR-214 suppresses the expression and mitochondrial translocation of Bim1 and increases the levels of phosphorylated Bad in cardiomyoblasts H9C2 cells H9C2 cells were transfected with miR-214 mimics. miR-control mimics served as control. The cells were subjected to hypoxia (4 h) followed by reoxygenation (24 h). The cells were harvested for isolation of mitochondria. The levels of Bim1 **A** and **B**. and phosphorylated Bad **C**. were examined by Western blot. n=3/group. **D**. Mitochondrial membrane potential was measured by JC-1 Dye (n=6-8). * p<0.05 compared with indicated groups. # p<0.05 compared with respective control.

To examine the effect of miR-214 on Bim1 translocation from cytosol to mitochondria, we transfected H9C2 cells with miR-214 mimics or scrambled miR-control respectively, before the cells were subjected to hypoxia/reoxygenation (H/R). Figures 5A and 5B show that H/R induces translocation of Bim1 from the cytosol to the mitochondria as evidenced by a significant decrease in the cytosolic levels of Bim1 (A), but a markedly enhanced amount of Bim1 in mitochondrial extracts (B). In contrast, miR-214 markedly suppresses the expression of Bim1 in the cytosol and decreases the levels of Bim1 in the mitochondria following H/R challenge. MiR-214 transfection also significantly increases Bad phosphorylation in the presence and absence of H/R compared with untransfected control groups (Figure 5C). Transfection of scrambled miR-control did not induce Bad phosphorylation in the presence and absence of H/R. In addition, H/R markedly decreased mitochondrial membrane potential which was significantly attenuated by LmiR-214 transfection (Figure 5D).

## DISCUSSION

In the present study, we demonstrated that increased expression of miR-214 in the myocardium significantly attenuates I/R-induced cardiac dysfunction and myocardial infarct size. The mechanisms involve an anti-apoptotic effect via activation of PI3K/Akt signaling and suppression of Bim expression. Specifically, we observed that miR-214 suppresses PTEN expression, leading to activation of PI3K/Akt signaling. It is well known that activated Akt phosphorylates Bad [13], thereby blocking the pro-apoptotic effect of Bad. In addition, miR-214 represses the expression of pro-apoptotic protein Bim1 [10] and its translocation from cytosol to the mitochondria, thus preventing I/R induced apoptosis.

We have previously reported that there is an interaction between TLR-mediated pathway and PI3K/Akt signaling during myocardial I/R injury [20, 37]. Activation of PI3K/Akt signaling has been reported to protect the myocardium from I/R injury and promotes cell survival following H/R [27, 28, 37, 38]. However, the mechanisms by which stimulation of TLRs induces activation of PI3K/Akt signaling are unclear. Interestingly, we observed that miR-214 expression in the myocardium was significantly increased by a TLR2 ligand, Pam3CSK4, in the presence and absence of I/R. Since Pam3CSK4 induced activation of PI3K/Akt signaling [20], we examined whether increased miR-214 expression would activate PI3K/Akt signaling. Indeed, our data show that increased expression of miR-214 by transfection of LmiR-214 into H9C2 cells suppresses PTEN expression and increases the levels of Akt phosphorylation in the presence and absence of H/R challenge. LmiR-214 transfection also markedly attenuates H/R-induced cell injury as evidenced by decreased LDH release after the cells were subjected to H/R. In addition, cell viability was markedly improved by increased expression of miR-214. Collectively, the *in vitro* data suggests that miR-214 has a protective effect on H/R-induced cell injury via suppression of PTEN, leading to activation of PI3K/Akt signaling.

Next, we investigated whether increased expression of miR-214 in the myocardium would protect the hearts from I/R-induced injury. We constructed LmiR-214 and transfected it into the myocardium through the right carotid artery seven days before the hearts were subjected to myocardial I/R [1, 2]. The *in vivo* data shows that LmiR-214 transfection prevents I/R-induced cardiac dysfunction and reduced I/R-induced myocardial infarct size. Our data is consistent with the report by Aurora *et al* [10] showing that deficiency of miR-214 resulted in more severe cardiac dysfunction and myocardial I/R injury, indicating that miR-214 is essential for cardioprotection against I/R injury. To understand the mechanisms by which miR-214 protects against myocardial I/R injury, Aurora *et al* [10] observed that miR-214 targets sodium-calcium exchanger-1, thereby influencing calcium trafficking in cardiac myocytes after myocardial I/R injury [10]. We found that miR-214 significantly suppresses PTEN expression, leading to Akt phosphorylation in the myocardium. Previous studies have demonstrated that activation of PI3K/Akt signaling plays a critical role in reduced I/R-induced injury and attenuated I/R-induced cardiac dysfunction [18–21]. Our data indicates that activation of PI3K/Akt signaling by miR-214 may be an important mechanism for protection against I/R injury.

We observed that increased expression of miR-214 significantly attenuates I/R-induced myocardial apoptosis. Apoptosis has been demonstrated to contribute to I/R-induced myocardial damage and cardiac dysfunction [31]. Apoptosis is the process of programmed cell death which is predominately dependent on mitochondrial function [39]. Bcl2 family members play a critical role in regulating mitochondrial mediated apoptosis through controlling the permeabilization of the mitochondrial membrane [39]. Bad is a pro-apoptotic protein that promotes activation of the programmed cell death pathway through formation of heterodimerizes with Bcl2 or Bcl-XL to inhibit their anti-apoptotic functions [35]. Our *in vitro* and *in vivo* data show that increased miR-214 expression significantly enhances Bad phosphorylation, thereby blocking Bad's pro-apoptotic effect. It has been reported that Bad is phosphorylated by a variety of kinases including Akt and P70S60 kinase [13, 34] with subsequent loss of its pro-apoptotic action by binding with 14-3-3 protein [35, 40]. Our findings indicate that targeting PTEN expression by mir-214, resulting in activation of Akt could be responsible for Bad phosphorylation.

We also found that increased expression of miR-214 represses the expression of Bim1 and prevents its translocation from the cytosol to mitochondria. Bim1 is another pro-apoptotic protein of Bcl-2 family and triggers apoptosis by inhibiting Bcl2 anti-apoptotic function and/or direct activation of Bax [14], leading to cytochrome c release and activation of apoptotic signaling [39]. Therefore, suppression of Bim1 expression and preventing its translocation from the cytosol to the mitochondria are necessary for preventing apoptosis during myocardial I/R injury [36]. Aurora *et al* reported that the expression of Bim1 in the myocardium was significantly increased in miR-214 deficient mice 7 days after myocardial I/R injury, indicating that miR-214 may target Bim [10]. We demonstrated in the present study that increased *in vivo* expression of miR-214 by LmiR-214 transfection suppresses the expression of Bim1 in the myocardium following I/R injury and that *in vitro* enhanced miR-214 levels prevent the translocation of Bim1 from the cytosol to the mitochondria. Collectively, targeting Bim1 expression and preventing its translocation from cytosol to mitochondria could be an important protective mechanism of miR-214 in myocardial I/R injury.

In summary, our data demonstrated that miR-214 plays a protective role in myocardial I/R injury. The mechanisms involve an anti-apoptotic effect through suppression of PTEN expression, leading to activation of PI3K/Akt signaling which suppresses Bad activity via its phosphorylation. In addition, miR-214 suppresses the expression and translocation of Bim1 from the cytosol to the mitochondria. MiR-214 could be a target for the induction of protection against myocardial I/R injury. Future studies should search for which natural conditions will induce miR-214 expression.

## MATERIALS AND METHODS

### Animals

Male C57BL/6J mice were obtained from Jackson Laboratory and maintained in the Division of Laboratory Animal Resources, East Tennessee State University (ETSU). The experiments outlined in this article conform to the Guide for the Care and Use of Laboratory Animals published by the National Institutes of Health (NIH Publication, 8^th^ Edition, 2011). All aspects of the animal care and experimental protocols were approved by the ETSU Committee on Animal Care.

### Induction of myocardial I/R injury

Myocardial I/R injury was induced as described previously [1, 2, 18, 20, 21]. Briefly, the mice were anesthetized by 5.0% isoflurane, intubated and ventilated using a rodent ventilator. Anesthesia was maintained by inhalation of 1.5% to 2% isoflurane driven by 100% oxygen flow. Body temperature was regulated at 37°C by surface water heating. The hearts were exposed and the left anterior descending (LAD) coronary artery was ligated with an 8-0 silk ligature. After completion of 45 min of occlusion, the coronary artery was reperfused by releasing the suture knot. After reperfusion for the time indicated, the mice were euthanized by CO_2_ inhalation and the hearts were harvested. Infarct size was evaluated by triphenyltetrazolium chloride (TTC, Sigma-Aldrich) staining as described previously [1, 2, 18, 20, 21].

### Echocardiography

Transthoracic two-dimensional M-mode echocardiogram and pulsed wave Doppler spectral tracings were obtained using a Toshiba Aplio 80 Imaging System (Tochigi, Japan) equipped with a 12-MHz linear transducer as described previously [1, 2, 19, 20]. Ejection fraction (EF) and percent fractional shortening (FS) were calculated as described previously [1, 2, 19, 20].

### In situ apoptosis assay

Measurement of cardiac myocyte apoptosis was performed as described previously [1, 2, 18–21] using the *in situ* cell death detection kit, fluorescein (Roche, USA) according to instructions of the manufacturer.

### Measurement of cell viability and mitochondrial membrane potential

Cell viability was assessed by measuring mitochondrial dehydrogenase activity using the MTT assay kit (Sigma). Cell injury was assessed by measurement of lactate dehydrogenase (LDH) activity in culture medium using a commercial kit (Cytotoxicity Detection Kit, Sigma). Mitochondrial membrane potential was evaluated by the fluorescence ratio of JC-1 aggregates (red) to monomers (green).

### Real time PCR assay of miRNAs

miRs were isolated from heart tissues or cultured cells using the miRNAs isolation kit (RNAzol®RT, MRC) in accordance with the manufacturer's protocol. Quantitative real-time (qPCR) was conducted using a 4800 Real-time PCR machine (Bio-Rad). MicroRNA levels were quantified by qPCR using specific Taqman assays for miR (Applied Biosystems, USA) and Taqman Universal Master Mix (Applied Biosystems). Specific primers for miR-214 were obtained from Applied Biosystems. MicroRNA-214 levels were quantified with the 2 (-DDct) relative quantification method that was normalized to the snRU6.

### Construction of lentivirus expressing miR-214

MiR-214 was constructed into a lentivirus expressing vector using a lentivirus expressing system (Invitrogen Corporation) as described previously [1, 2]. Briefly, the oligonucleotides for miR-214 were synthesized at Integrated DNA Technologies, annealed and ligated into pcDNATM6.2-GW/EmGFP-miR. The pcDNATM6.2-GW/EmGFP-miR cassette was subsequently transferred to pDONR221TM and finally pLenti6/V5-DEST by two sequential Gateway BP and LR recombinations. The construct was verified by sequencing. The viral particles were produced by third generation packaging in 293FT cells and lentiviral stocks were concentrated using ultracentrifugation.

### Transfection of lentivirus expressing miRNA

We transfected mouse hearts with lentivirus expressing miR-214 (LmiR-214) or lentivirus expressing miR-control (LmiR-control) via the right common carotid artery as described previously [1, 2]. Briefly, mice were intubated and anesthetized with mechanical ventilation using 5% isoflurane. The anesthesia was maintained by inhalation of 1.5-2% isoflurane in 100% oxygen. An incision was made in the middle of the neck and the right common carotid artery was carefully isolated. A micro-catheter was introduced into the isolated common carotid artery and positioned into the aortic root through an arteriotomy site in the external carotid artery [1, 2]. One hundred microliters of LmiR-214 (1×10^7^ PFU) or LmiR-Con was injected through the micro-catheter. The micro-catheter was gently removed and the common carotid artery was tightened before the skin was closed. Seven days after transfection, the hearts were harvested and transfection efficiency was evaluated by examining the green fluorescent protein (GFP) expression and the expression of miR-214 in the heart tissues.

### *In vitro* experiments

The H9C2 cardiomyoblasts stably expressing miR-214 or miR-con were generated by transfection of LmiR-214 or LmiR-con and selection with Blasticidin (Invitrogen). Transient expression of miR-214 in H9C2 cells was accomplished by transfection of miR-214 mimics (40 nM) or miR-control mimics (40 nM). The cells were subjected to hypoxia/reoxygenation as described previously^1^. Briefly, the medium was changed to hypoxia-equilibrated medium immediately before the cells were incubated at 37°C with 5% CO2 and 0.1% O2 in a hypoxia chamber (Pro-Ox Model C21, BioSpherix Ltd, Redfield NY) for 4 h followed by reoxygenation in an incubator with 5% CO_2_. The cells that were not subjected to H/R served as control (normoxia). The cells were harvested and cellular proteins were isolated for Western blot analysis [19–21].

### Mitochondrial isolation and Western blot

Mitochondria were isolated from H9C2 cells using a mitochondrial isolation kit (Thermo Scientific) according to the manufacturer's protocol. Western blots were performed as described previously [19–21]. The membranes were incubated with appropriate primary antibodies respectively, including anti-PTEN, anti-p-Akt, anti-Akt, anti-Bim, anti-p-Bad, anti-Bad (Cell Signaling Technology, Inc, Danvers, MA), respectively, followed by incubation with peroxidase-conjugated second antibodies (Cell Signaling Technology, Inc.) and examination with the ECL system (Amersham Pharmacia, Piscataway, NJ). The signals were quantified using a G: Box gel imaging system by Syngene (Syngene, USA, Frederick, MD).

### Caspase-3/7 and caspase-8 activities assay

Caspase-3/7 and caspase-8 activity were measured using a Caspase-Glo assay kit (Promega) according to the manufacturer's protocol as described previously [20].

### Statistical analysis

Data are expressed as mean ± SD. Comparisons of data between groups were made using one-way analysis of variance (ANOVA), and Tukey's procedure for multiple-range tests was performed. The overall survival probabilities were analyzed using the log-rank test. P< 0.05 was considered to be significant.
